# Case Report: Self-Administration of Omalizumab in an Adolescent With Severe Asthma During SARS-CoV-2 Infection

**DOI:** 10.3389/fped.2021.675281

**Published:** 2021-12-06

**Authors:** Erika Paladini, Mattia Giovannini, Simona Barni, Giulia Liccioli, Lucrezia Sarti, Elio Novembre, Francesca Mori

**Affiliations:** ^1^Pediatric Respiratory and Allergy Unit, Women's and Children's Health Department, University of Padua, Padua, Italy; ^2^Allergy Unit, Department of Pediatrics, Meyer Children's University Hospital, Florence, Italy

**Keywords:** allergy, asthma, omalizumab, clinical practice, COVID-19, pediatrics

## Abstract

Acute asthma remains one of the most frequent causes of children's access to healthcare. Asthma exacerbation is an essential defining characteristic of its severity, and respiratory infections entail increased risks of exacerbations with potential hospitalization. In the literature, contradictory findings have been reported about the risk and severity of severe acute respiratory syndrome coronavirus 2 (SARS-CoV-2) infection in patients affected by asthma, with several implications for its management. Anti-IgE monoclonal antibody therapy is meant for patients affected by severe persistent allergic asthma without adequate control with other treatments. Indeed, biological therapies, such as omalizumab, are used as add-on treatments (step 5 in the Global Initiative for Asthma report) for severe asthma with several benefits, including a reduction in the frequency of exacerbations. To the best of our knowledge, we hereby report the first case in which an adolescent with severe allergic asthma treated with omalizumab has switched to self-administration at home during SARS-CoV-2 infection. Based on our peculiar experience, physicians may consider switching to self-administration of omalizumab at home, even during the coronavirus disease 2019 pandemic. However, more extensive research data from future studies are needed to confirm these first findings.

## Introduction

Asthma is a heterogeneous disease usually characterized by chronic airway inflammation. Dyspnea, wheezing, cough, and chest tightness are the main signs and symptoms. Acute exacerbations of asthma are an important treatment challenge consequently leading to hospital admission (1). Asthma affects about 13.7% of children aged 13–14 years and 11.6% aged 6–7 years worldwide (2). The international European Respiratory Society/American Thoracic Society guidelines classify the severity of asthma by the type of treatment used by the patients to control its clinical manifestations (3). Exacerbations are considered the key to characterize asthma severity, and their prevention is an important parameter to evaluate asthma treatments (4).

In December 2019, coronavirus disease 2019 (COVID-19) infection, an acute respiratory illness, occurred in Wuhan, Hubei Province, China, and rapidly spread worldwide (5–8). The World Health Organization declared severe acute respiratory syndrome coronavirus 2 (SARS-CoV-2) a public health emergency on January 3, 2020 (9–13). At the beginning, the hypothesis was that those patients affected by asthma would have been at increased risk of COVID-19 and its severe clinical manifestations (14). Nowadays, there are controversial hypotheses about the complex interaction of COVID-19 with asthmatic patients. From the pathophysiological point of view, it has been demonstrated that SARS-CoV-2 uses angiotensin-converting enzyme 2 (ACE2) as a cellular receptor. Decreased ACE2 expression is found in the respiratory epithelial cells of patients with allergy and asthma and may protect against COVID-19 infection and severe clinical manifestations (15). However, in asthmatic patients, there is a reduction of the antiviral immune response and an important risk of exacerbations caused by the virus, potentially leading to increased susceptibility to and severity of SARS-CoV-2 infection (15–17). Thus, it is advised that asthmatic patients continue with their therapy during the COVID-19 pandemic (17). Such recommendation also applies to monoclonal antibody therapy to manage asthma. Indeed, biological therapies, such as omalizumab, are used as an add-on treatment [step 5 in the Global Initiative for Asthma (GINA) report] (4) for severe asthma with several benefits, including a reduction in the frequency of exacerbations (18). Omalizumab can be prescribed to address severe persistent allergic asthma in patients over 6 years old. Specifically, in adults and adolescents, 12 years old and above, the latter drug is indicated as an add-on therapy in patients with a positive skin test or *in vitro* reactivity to a perennial aeroallergen, reduced lung function (FEV_1_ < 80%), and frequent daytime clinical manifestations or night-time signs and symptoms with repeated severe asthmatic exacerbations. Its ease of handling, efficacy, and safety should encourage patient self-administration of omalizumab at home (19). Appropriate training sessions performed by a physician are needed to help and support biological therapy switch from hospital to home self-administration in order to prevent complications and adverse reactions (20).

## Case Report

We hereby report the case of a 16-year-old Caucasian female who has been followed at our Allergy Unit of Meyer Children's University Hospital in Florence for allergic asthma since the age of 6. At the physical examination, she presented a history of respiratory clinical manifestations such as cough and shortness of breath. During her asthma history, she also reported two hospitalizations: the first one during a wheezing episode triggered by an airway infection and the second one during an asthmatic attack without an infection. She required treatment with short-acting β2-agonist and systemic corticosteroids during her asthma exacerbations. The patient presented positive skin prick tests to house dust mites and cat fur from the first clinical evaluation and a positive skin prick test to pollen (grass, mugwort, hazel, birch, and poplar) during the follow-up.

She also suffered from food allergy, i.e., to nuts, with sensitization to lipid transfer protein and profilin. At the age of 2, the patient had anaphylaxis after eating cashew and adrenaline autoinjectors were prescribed. She had skin prick tests, prick by prick tests, and blood tests for nuts, and they resulted positive not only for cashew but also for peanut, almond, hazelnut, walnut, pine nut, and pistachio, which were all excluded from the diet. Moreover, with carrots and fennels, she presented itch in her throat and dyspnea. For this reason, following the positive skin prick tests, the patient also excluded these foods from the diet. At 16 years old, the patient presented anaphylaxis twice after eating a pear and shrimps, which were then excluded from her diet.

Apart from asthma and food allergy, she did not suffer from other illnesses. The patient reported a parental history of atopic disease: her mother suffered from nickel contact allergy and her father from rhinoconjunctivitis with grass and *Parietaria* pollen sensitization.

We have evaluated all the possible differential diagnoses with asthma or additional factors, which were eventually ruled out. For example, no clinical features of chronic bronchitis, cystic fibrosis, or gastroesophageal reflux were detected. The patient also underwent an electrocardiogram, which did not reveal any rhythm abnormalities.

**Figure 1:**
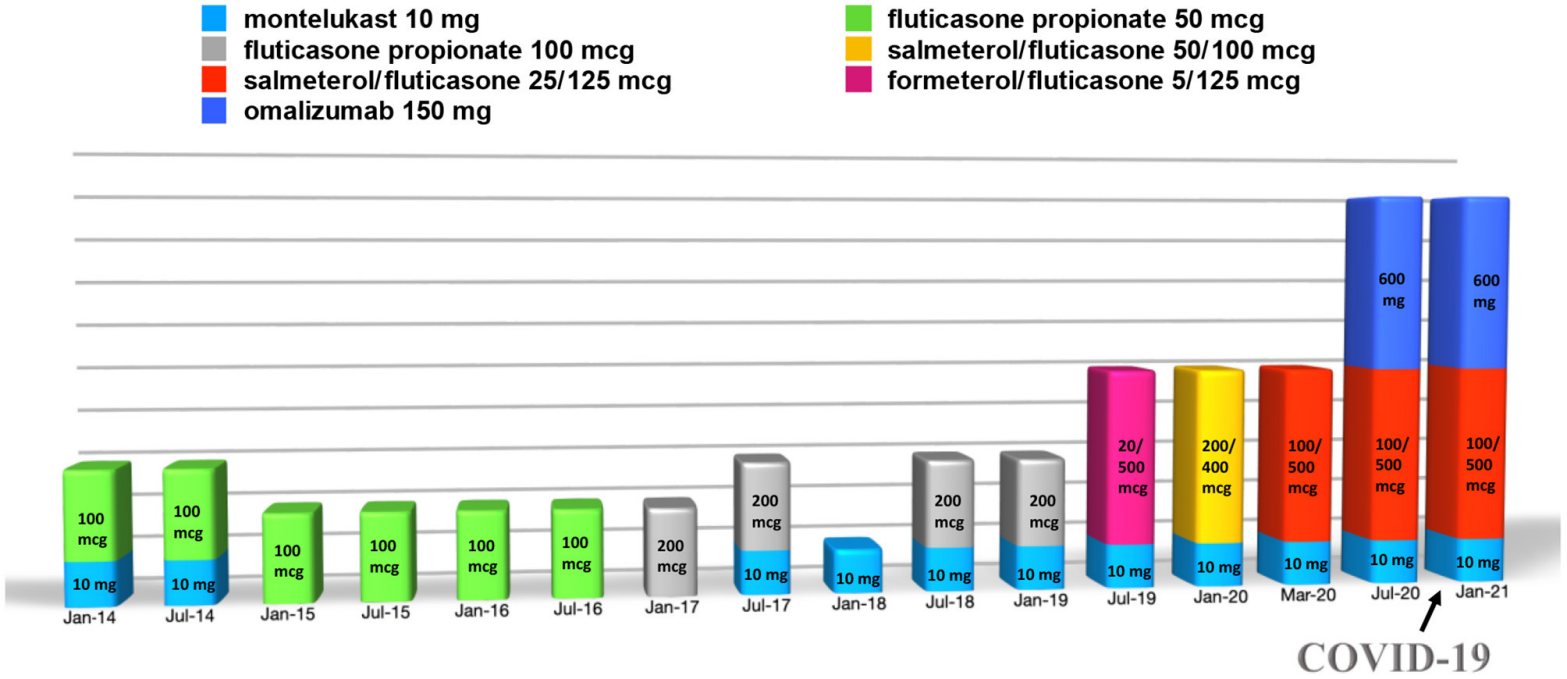
Summary of the treatment carried out by the patient over time (doses of the drugs are intended as daily and of omalizumab as every 2 weeks).

After the diagnosis of asthma, she attended periodic follow-up visits at our Allergy Unit, where spirometry was performed each time as well. Afterwards, at 16 years old, her asthma clinical manifestations worsened progressively, becoming severe despite treatment with high-dose inhaled corticosteroid, long-acting β2-agonist, and anti-leukotriene (fluticasone/salmeterol and montelukast) (Figure 1). Indeed, she had frequent asthma exacerbations, especially in the evenings, about once every month, and dyspnea for minimal physical efforts while under these treatments. In addition, the patient presented a spirometry with a reversible lung obstruction. Indeed, the patient presented a basal FEV_1_ of 79% with a positive bronchodilatation test equal to 290 ml (+12%).

**Figure 2:**
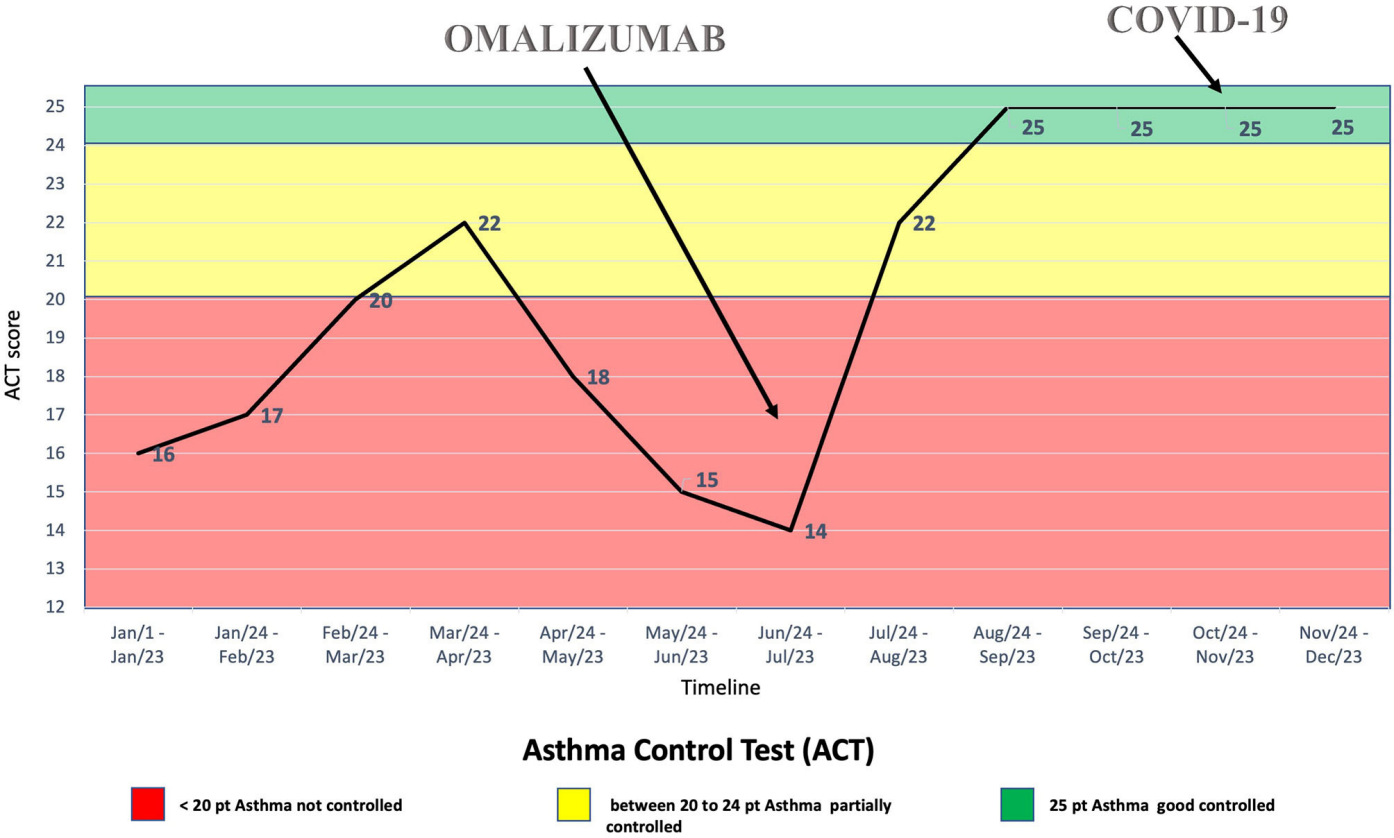
Summary of the Asthma Control Test recorded with the patient over time.

Thus, treatment with subcutaneous injections of the anti-IgE antibody omalizumab, 600 mg every 2 weeks, was started at the age of 16 years, although it was used as off-label due to her high total IgE serum concentration (2,003 kU/L). The patient's clinical condition benefitted from the treatment with omalizumab (Figures 1, 2), with clinical improvements after the first injection and with an improvement of the spirometry (FEV_1_ = 94%, with a negative bronchodilatation test) performed after the seventh injection.

During the SARS-CoV-2 pandemic, the patient did not change her habits, including going to school, and on October 20, 2020, one of her classmates resulted positive for SARS-CoV-2 from real-time polymerase chain reaction (RT-PCR) on a nasopharyngeal swab. Therefore, she was sent home for quarantine. Indeed, she did not suffer from clinical manifestations typical of COVID-19, nor her asthmatic signs or symptoms did worsen at the time. However, after 7 days, she performed RT-PCR on a nasopharyngeal swab, which resulted positive for SARS-CoV-2. The patient was still without clinical manifestations at the time, but after 2 days she developed diarrhea, asthenia, myalgia, epistaxis, and maximum body temperature 37.5°C treated successfully with paracetamol. At that time, the patient had been undergoing therapy with subcutaneous omalizumab every 2 weeks for about 3 months, as well as fluticasone/salmeterol and montelukast daily (Figure 1). Moreover, during quarantine, omalizumab treatment was easily continued at home, and it was switched to self-administration through telephonic support and digital material available online (21), such as educational videos and the Asthma Control Test. It is worth mentioning that the patient remained free of asthma clinical manifestations the whole time she was positive of SARS-CoV-2, without significant differences in asthma management during this period (Figure 2). Furthermore, no drug adverse events have been recorded. Finally, she reported good self-confidence with the administration of omalizumab at home.

## Discussion

Omalizumab is a humanized monoclonal IgG1 antibody that binds the free circulating IgE and prevents the interaction of the latter with mast cells and the consequent release of inflammatory molecules (22–24). Furthermore, it determines a downregulation of FcεRI (the high-affinity Fc region IgE receptor) expression on basophils and mast cells (25). Omalizumab also reduces the *in vivo* expression of FcεRI on dendritic cells (26) and the activation of eosinophils preventing allergic inflammation (27, 28).

Omalizumab is administered subcutaneously. The dosage and the frequency of injection are established according to the total serum IgE level (30–1,500 kU/L) and the patient's weight (29). Omalizumab is usually administered in a hospital setting because the patient should be monitored after the injection, especially at the beginning of the treatment. Many studies have shown the safety and efficacy of this biological drug (30–36), also in pediatric patients, through clinical trials and real-life experiences (31, 33, 34, 36). Omalizumab has also been used successfully in patients with seasonal asthmatic exacerbations during the autumn and spring seasons (36, 37) with total IgE serum values >2,000 kU/L (38) and in patients with severe intrinsic asthma (39). Headache and redness, swelling, pain, or itching on the site of injection are the main adverse reactions reported among patients aged 12 years (29). However, among the biological drugs available, omalizumab is the one with the most significant data on efficacy and safety, which come from clinical trials and real-life clinical data (1).

COVID-19 represents a public health emergency (13, 16). For this reason, attention should be paid to the treatment management of patients with chronic underlying diseases, including those with asthma. Indeed, asthma exacerbations may be determined by different factors such as respiratory infections, air pollutants, or inadequate adherence to the treatment.

Asthmatic patients were considered to have a potentially increased susceptibility to and severity of SARS-CoV-2 resulting from a decrease in antiviral immune response (15–17). However, in the literature, there is evidence that patients affected by asthma may have a lower risk of COVID-19, including its severe forms. Indeed, some published experiences have shown that only a low percentage of hospitalized adult patients with COVID-19 was affected by asthma (40, 41). This may be explained by the fact that the ACE2 which is important for the uptake of SARS-CoV-2, is downregulated in the respiratory airway of patients affected by asthma and allergies (15). Apart from this, contradictory findings have been reported on the risk and severity of SARS-CoV-2 infection in adult patients affected by asthma (42). Concerning the pediatric population, Ciprandi et al. have reported that only one (2%) young patient hospitalized for COVID-19 presented asthma in the previous clinical history out of 52 young patients hospitalized for COVID-19 in two hub hospitals covering the regions of South Lombardy and Liguria (43). On the other hand, contradictory findings have been reported on the risk and severity of SARS-CoV-2 infection in pediatric patients affected by asthma (44). However, these data underline how limited our current experience with the pediatric population is on this specific topic.

In addition, it has been speculated that treatments with the anti-IgE antibody omalizumab, in a patient with severe allergic asthma, may protect from the severe forms of COVID-19. Indeed, omalizumab increases the antiviral immune response by downregulating the high-affinity IgE receptor on plasmacytoid dendritic cells (45, 46). However, this has not been explicitly demonstrated in reference to SARS-CoV-2 infection, and future studies will be needed to shed light on this specific potential effect in the latter case. Taking into consideration all these elements, we speculate that the patient described in our case report might have been protected against exacerbations of allergic asthma during COVID-19, also thanks to the omalizumab therapy. However, more solid evidence from future studies is needed to confirm our hypothesis.

Lommatzsch et al. have reported a case of a 52-year-old man with severe allergic asthma treated with omalizumab during his home quarantine period after SARS-CoV-2 infection. During that time, he continued his self-administered therapy with omalizumab at home without asthma deterioration or therapy complications. As previously proposed above, in the interpretation of their experience, the authors suggested that patients with allergic asthma may have a lower risk of COVID-19 and that the anti-IgE antibody, i.e., omalizumab, may improve the antiviral immune response (47). In this regard, Hanon et al. have analyzed a cohort of 676 adult patients with severe asthma, 129 of whom were on therapy with omalizumab. A small number of COVID-19 cases were found, none of which died or had a very severe disease course. These data have proven the importance of continuing with biologic treatments in severe asthma during the COVID-19 pandemic (48). However, specific data on the topic are scarce in pediatric patients, and future studies will be needed to shed light on this.

In conclusion, to the best of our knowledge, we hereby report the first case in which an adolescent with severe allergic asthma treated with omalizumab switched to its self-administration at home during SARS-CoV-2 infection. It is essential to know that managing patients with severe asthma during the COVID-19 pandemic might be challenging, particularly in children on therapy with biologics. Thus, any possible effort should be made to control the asthmatic disease. For these reasons, monoclonal antibody therapy such as omalizumab could be reasonably continued in severe asthmatic patients during the pandemic to protect them from exacerbations (43, 48). Based on our peculiar experience, physicians may consider switching to self-administration of omalizumab at home, even during the COVID-19 pandemic. However, more extensive research data from future studies are needed to confirm these first findings.

## Data Availability Statement

The original contributions presented in the study are included in the article/supplementary material. Further inquiries can be directed to the corresponding author.

## Ethics Statement

Written informed consent was obtained from the minor(s)' legal guardian/next of kin for the publication of any potentially identifiable images or data included in this article.

## Author Contributions

FM conceptualized the work. EP, MG, EN, and FM drafted the manuscript. EP, MG, SB, GL, LS, EN, and FM performed the investigations and critically revised the manuscript. All authors contributed to the article and approved the submitted version.

## Funding

The publication fee was financed by Novartis. The funder was not involved in the study design, collection, analysis, interpretation of data, the writing of this article, or the decision to submit it for publication.

## Conflict of Interest

The authors declare that the research was conducted in the absence of any commercial or financial relationships that could be construed as a potential conflict of interest.

## Publisher's Note

All claims expressed in this article are solely those of the authors and do not necessarily represent those of their affiliated organizations, or those of the publisher, the editors and the reviewers. Any product that may be evaluated in this article, or claim that may be made by its manufacturer, is not guaranteed or endorsed by the publisher.
